# Noninvasive Assessment of the Severity of Liver Fibrosis in MASLD Patients with Long-Standing Type 2 Diabetes

**DOI:** 10.1007/s11606-025-09348-2

**Published:** 2025-01-22

**Authors:** Farooq Khan, Stafny Dsouza, Amar Hassan Khamis, Fatima Abdul, Muhammad Hamed Farooqi, Fatima Sulaiman, Fahad Mulla, Fatheya Al Awadi, Mohammed Hassanein, Riad Bayoumi

**Affiliations:** 1 Hepatology, King’s College Hospital London, Dubai, United Arab Emirates; 2 College of Medicine, Mohammed Bin Rashid University of Medicine and Health Sciences, Dubai, United Arab Emirates; 3 Hamdan Bin Mohammed College of Dental Medicine, Mohammed Bin Rashid University of Medicine and Health Sciences, Dubai, United Arab Emirates; 4 Dubai Diabetes Center, Dubai Health, Dubai, United Arab Emirates; 5 Endocrinology Department, Dubai Hospital, Dubai Health, Dubai, United Arab Emirates

**Keywords:** Nonalcoholic fatty liver disease, Metabolic dysfunction–associated steatotic liver disease, Type 2 diabetes, Emirati population, Fibrosis, Serum biomarkers, Inflammation

## Abstract

**Background:**

Type 2 diabetes mellitus (T2DM) and metabolic dysfunction–associated steatotic liver disease (MASLD), which have a reciprocal relationship compounded by obesity, are highly prevalent in the Middle East affecting morbidity, mortality, and healthcare costs.

**Objective:**

This study aimed to assess the severity of MASLD and liver fibrosis among adult Emirati patients with long-standing T2DM.

**Design and Participants:**

This cross-sectional study used noninvasive methods to assess the severity of MASLD and fibrosis progression in an adult cohort of Emirati patients (*N* = 546) with a mean T2DM duration of 16 years.

**Main Measures:**

Fatty liver infiltration was assessed by hepatic steatosis index (HSI), while fibrosis was assessed by the fibrosis-4 (FIB-4) index and aspartate aminotransferase/platelet ratio index (APRI). Of those, 108 patients were randomly subjected to ultrasound-based FibroScan^®^ to assess controlled attenuation parameter (CAP) and liver stiffness measurement (LSM).

**Key Results:**

All patients had fatty liver with ~ 83% being categorized as having severe steatosis. Serum-based fibrosis biomarker panels detected significant liver fibrosis in ~ 2.5% of these patients. The APRI appeared to be more restrictive in detecting moderate fibrosis (1.5%) than the FIB-4 index (25.5%). CAP significantly correlated with the LSM, indicating that the two methods contributed to the same underlying pathophysiology. Liver steatosis was more severe in female patients, who were older and had a higher body mass index (BMI) than those with moderate or no significant fibrosis. They also had higher serum liver enzymes and were more likely to have age-related changes in kidney function. Interestingly, severity of both steatosis and fibrosis remained unaffected by age and duration of T2D except for fibrosis severity detected by FibroScan^®^.

**Conclusions:**

This study highlights the critical need for routine screening of MASLD among Emirati patients with long-standing T2DM, given the high point prevalence of severe steatosis (~ 83%), predominantly among women in this population.

**Supplementary Information:**

The online version contains supplementary material available at 10.1007/s11606-025-09348-2.

## INTRODUCTION

Nonalcoholic fatty liver disease (NAFLD) is a spectrum of progressive liver disorders characterized by hepatic steatosis on imaging or histology (macro-vesicular steatosis) and the absence of secondary causes of hepatic steatosis, such as significant alcohol consumption, chronic use of medications that cause hepatic steatosis, or hereditary disorders. It is a rapidly growing public health concern affecting approximately one-fourth of the global population, more so among men than women.^1–3^

A Delphi consensus by the American Association for Study of Liver Disease, the European Association for Study of the Liver, and the Asociación Latinoamericana para el Estudio del Hígado has now changed the nomenclature of NAFLD to metabolic dysfunction–associated steatotic liver disease (MASLD).^4,5^ MASLD is the liver manifestation of metabolic syndrome closely associated with obesity, dyslipidemia, type 2 diabetes mellitus (T2DM), and hypertension.^6,7^ Due to the establishment of a 99% overlap between MASLD- and historical NAFLD-defined populations, findings from NAFLD studies remain valid under the new MASLD definition.^8–10^

Despite affecting 25–32% of the pooled global adult population, only a minority of patients with MASLD develop metabolic dysfunction-associated steatohepatitis (MASH), which progresses to significant fibrosis.^3,11,12^ The progressive nature of MASLD, particularly when associated with advanced fibrosis, needs to be identified in patients at risk (aged > 50 years, with T2D or metabolic syndrome) because of its prognostic implications.^13^ In an epidemiological cross-sectional study involving 500 participants, 10.2% were diagnosed with MASLD alone and had a greater risk of liver fibrosis and disease progression.^14^ T2DM, a main criterion for MASLD classification, has a reciprocal relationship with MASLD compounded by obesity.^15^ The complex interrelationship between these conditions is driven by several pathophysiological pathways including insulin resistance,^16^ inflammation,^17^ and lipotoxicity.^18,19^ T2DM is both a consequence and a contributor to MASLD as hyperglycemia exacerbates liver inflammation and fibrosis. Chronic inflammation also plays a central role in the pathogenesis of MASLD, with pro-inflammatory cytokines and oxidative stress further damaging liver cells.^17,19^ Excess adipose tissue in obesity contributes to insulin resistance which in turn, promotes hepatic fat accumulation and inflammation, leading to MASLD.^19^ This interplay underscored the importance of addressing the association of T2DM and MASLD in our studies.

T2DM has a significant impact on morbidity, mortality, and healthcare costs.^20–23^ It affects 55.5% of the global population with MASLD and 37.3% with MASH.^24^ Certain ethnic groups, particularly those of Asian and Middle Eastern origin, are considered at risk mainly because of changing lifestyles, dietary habits, and cardiovascular morbidity and may result in a major healthcare burden, mainly in Southeast Asia, North Africa, the Middle East, and southern sub-Saharan Africa.^25–27^ A meta-analysis of adult patients with MASLD reported a higher prevalence in the Middle East region (31.79%) than in other regions.^2^ As of 2017, approximately 25% of the estimated Emirati population of the UAE was diagnosed with MASLD, and the prevalence of MASLD is projected to double in the next decade.^28^

The clinical and economic limitations of liver biopsy, the “gold standard” for diagnosing liver disease, have prompted the establishment of noninvasive tests (NITs), such as serum biomarkers, scoring systems, and imaging tests, to differentiate steatohepatitis from simple steatosis and to identify fibrosis, as it is the critical determinant of progression/regression, prognosis, and treatment decisions.^29–32^ Recent data suggest that the results from biomarker validation studies among patients with NAFLD can be applied to patients with MASLD.^11,33^ Lee et al.^34^ showed that the hepatic steatosis index (HSI), which uses liver enzyme levels and BMI for the detection of MASLD, is significantly correlated with hepatic steatosis grades determined by ultrasonography. The blood-based fibrosis-4 (FIB-4) index and AST:platelet ratio index (APRI) have also been proven to be reliable methods for detecting advanced fibrosis among MASLD patients with FIB-4 showing the best sensitivity and APRI showing the best specificity in predicting advanced stages of liver fibrosis among MASLD patients.^35^

Ultrasound-based vibration-controlled transient elastography (VCTE™: FibroScan^®^) is the most used noninvasive method for assessing liver stiffness and can be used to exclude significant hepatic fibrosis. A widely followed approach harnesses the potential of serum-based biomarkers and scoring systems in conjunction with liver stiffness measurement (LSM) by VCTE™ to assess the stage of fibrosis and portal hypertension in patients with chronic liver disease of different etiologies.^30–32,36^ The new Practice Guidance from the American Association for the Study of Liver Disease (AASLD)^37^ suggests the use of VCTE™ for detecting and diagnosing MASLD in high-risk individuals, including those with T2DM. It also suggests the use of the controlled attenuation parameter (CAP)™ (FibroScan^®^) as a point-of-care technique to identify steatosis, and if the FIB-4 index is ≥ 1.3, VCTE™ may be used to exclude advanced fibrosis.

According to the current consensus of the American Diabetic Association (ADA), in the absence of alcohol intake, patients with T2DM with either elevated liver enzymes or fatty liver on ultrasound imaging should be screened for steatohepatitis or fibrosis.^38^ Earlier, a meta-analysis by Younossi et al.^24^ reported a regional prevalence of 67.29% for MASLD among patients with T2DM in West Asia (Iran, Turkey, Saudi Arabia). Given the rapid increase in T2DM incidence in the Emirati population of the UAE^39^ and the resulting complex web of metabolic disturbances, it is crucial to screen for MASLD and associated complications in this population. Therefore, the primary aim of this study was to evaluate the severity of MASLD and liver fibrosis among Emirati patients with long-standing T2DM.

## MATERIALS AND METHODS

This cross-sectional study was conducted at Dubai Diabetes Center (DDC) and Dubai Hospital (DH), both of which follow the American Diabetes Association Standards of Medical Care for Diabetes.^40^ Ethical approval was obtained from the Dubai Scientific Research Ethics Committee (DSREC) and the Mohammed Bin Rashid University of Medicine & Health Sciences’ Institutional Review Board (MBRU-IRB). All participating patients were provided with detailed explanations of the study, reasons, and implications prior to obtaining written informed consent.

### Patients

A total of 623 Emirati patients who visited the DDC and DH between 2019 and 2022 were primarily screened for T2DM according to the following inclusion/exclusion criteria:

1. Patients aged > 18 years who provided written informed consent were included. Those who were not willing or unable to provide informed consent were excluded.
2. Patients were diagnosed with T2DM with or without complications by a registered healthcare practitioner according to the “SALAMA,” an Electronic Dubai Health Information System.
3. Patients must have a negative glutamic acid decarboxylase antibody (GADA) test.
4. Patients with type 1 diabetes mellitus (GADA test is positive), suspected maturity-onset diabetes of the young (MODY), or chronic diseases such as malignancy, blood diseases, or recent chemotherapy were excluded.

### Data Collection

Clinical data of the study participants were obtained by a healthcare practitioner through structured face-to-face questionnaires and supplemented by demographic information, medical history, lifestyle habits, and anthropometric measurements downloaded from the electronic SALAMA Health Information System, adhering to Dubai Health regulations and guidelines and conformed to the provisions of the Declaration of Helsinki (as revised in Fortaleza, Brazil, October 2013). The anonymized data included laboratory data comprising liver function tests, lipid profiles, fasting glucose levels, and hemoglobin A1c (HbA1c) levels.

Of the 623 T2D Emirati patients, 546 had complete data and satisfied the inclusion/exclusion criteria. The 546 patients were screened for steatosis by the HSI^34^ and fibrosis severity using the well-validated serum biomarker scoring systems FIB-4 index^41^ and APRI^42^ with the following formulae:

1. HSI = 8 × (ALT/AST ratio) + BMI + 2 (if female) + 2 (if *diabetes mellitus*)
2. APRI = (AST/ULN of AST = 40)/(PLT/ULN of PLT = 450,000)
3. FIB-4 = (age × AST)/(PLT × sqrt (ALT))

where AST is aspartate aminotransferase in U/L, ALT is alanine aminotransferase in U/L, BMI is body mass index in kilograms per square meter, ULN of AST is the upper limit of normal for AST, PLT is the platelet count in 10^9^/L, ULN of PLT is the upper limit of normal for platelet count, and age is in years. The severity was scaled according to the cut-offs shown in Tables 1 and 2.

**Table 1:** Cutoffs for the Categorization of Liver Steatosis Grades by Noninvasive Techniques

Grades	Grade 0	Grade 1	Grade 2	Grade 3
Interpretation	Mild steatosis		Moderate steatosis	Severe steatosis
Serum-based steatosis biomarker panels				
HSI^34^	< 30		≥ 30–36	≥ 36
Imaging-based steatosis detection				
CAP™ (dB/m)^43^	≥ 240–260		≥ 260–290	≥ 290–400
Assessment	Low risk		Intermediate	High risk

*HSI*, hepatic steatosis index; *CAP*, controlled attenuation parameter

**Table 2:** Cutoffs for the Categorization of Liver Fibrosis Severity by Noninvasive Techniques

Stages	F0	F1	F2	F3	F4
	Normal liver	Mild fibrosis	Moderate fibrosis	Severe fibrosis	Liver cirrhosis
Interpretation	No significant fibrosis		Intermediate fibrosis	significant fibrosis	
Serum-based fibrosis biomarker panels					
APRI^35^	< 0.5		≥ 0.5–0.702	≥ 0.702	
FIB-4^44^	< 1.30		≥ 1.30–2.67	≥ 2.67	
Imaging-based fibrosis detection					
LSM (kPa)^43^	< 8		≥ 8.0–12.0	≥ 12	
Assessment	Low risk		Uncertain	High risk	

*APRI*, aspartate aminotransferase-to-platelet ratio index; *FIB-4*, fibrosis-4 index; *LSM*, liver stiffness measurement

Upon receiving the informed consent, 108 patients from the study cohort, with similar clinical and laboratory parameters, were randomly selected on the basis of FIB-4 index (17% of low risk, 25% of uncertain, and 43% of high risk) and examined for LSMs expressed in kilopascals (kPa) by liver elastography (FibroScan^®^ compact 530; Echosens, Paris, France) and related to fibrosis severity. Simultaneously, the CAP™ score, a measure of fat accumulation in the liver expressed as decibels per meter (dB/m), was also determined from FibroScan^®^. Imaging was performed at the DDC between December 2022 and March 2023 by a trained FibroScan^®^ operator. Elastography was performed twice to obtain an average of two readings and reduce test bias. Table 1 and Table 2 show the cutoff values applied for determining steatosis and fibrosis risk, respectively.

### Statistical Analysis

Statistical data analysis was performed using IBM-SPSS for Windows version 28.0 (SPSS Inc., Chicago, IL). Categorical variables were described by using proportions, and continuous variables were described by a measure of tendency and a measure of dispersion. Categorical variables were cross-tabulated to examine the independence between variables; for such variables, the chi-square (*χ*^2^) test or Fisher’s exact test, as appropriate, was used. The Kolmogorov‒Smirnov test was used to test the normality of continuous variables. The Mann‒Whitney test was used to compare the means between two groups. Continuous variables are shown as medians (25th percentile, 75th percentile) and were compared using the Kruskal–Wallis test. To measure the linear and nonlinear relationships between two variables when the data were not normally distributed, the nonparametric Spearman’s rank correlation coefficient (*ρ*) was used. The diagnostic performance (positive predictive value (PPV), negative predictive value (NPV), sensitivity, and specificity) of serum-based markers against liver elastography methods for liver steatosis and fibrosis was categorically determined by receiver operating characteristic (ROC) curve analysis. A two-tailed *P* value < 0.05 was considered to indicate statistical significance in all analyses.

## RESULTS

### Clinical Characteristics of the Study Cohort

A total of 546 patients satisfied the inclusion–exclusion criteria of the study, with the majority being women (*N* = 311, 56.9%). The patients in the study cohort (*N* = 546) were all diagnosed with T2DM by registered medical practitioners in Dubai, with a mean duration of diabetes of 15.45 (± 8) years. The mean age at diagnosis was 42 (± 10) years, the mean fasting blood glucose level was 144 (± 51) mg/dL, the mean AST:ALT ratio was 1.09, the mean HbA1c level was 7.6 (± 1.6%), and the mean C-Reactive Protein was 17.1 mg/L (± 17.4 SD) (< 5 mg/L). No significant sex difference was observed (Table S1). The cohort included 0.7% underweight (*N* = 4), 11.1% normal (*N* = 61), 59.8% overweight (*N* = 180), and 55.1% obese (*N* = 301) participants. A total of 30% (*N* = 169) had elevated triglycerides, 5% (*N* = 29) had elevated AST, and 12% (*N* = 66) had elevated ALT. High waist circumference (WC) was reported in 60% of men (WC > 40 inches, *N* = 141/235) and 84% of women (WC > 35 inches, *N* = 262/311). Similarly, 62% of the patients had an HbA1c > 7% (Table S2).

### Hepatic Steatosis Severity Among Emirati Patients with MASLD

On screening for hepatic steatosis using serum HSI, 83% of the patients had significantly severe steatosis, and 15.4% had moderate steatosis (Table 3). There were no significant differences in age at diagnosis of T2DM or duration of diabetes among the three categories of steatosis. However, liver steatosis increased significantly (*P* < 0.001) with increasing BMI. Approximately 55% of the cohort were obese with severe steatosis (Supplementary Fig. 1).

**Table 3:** Demographics of 546 T2DM Emirati Patients Categorized Based on Steatosis Severity

Steatosis severity	Mild	Moderate	Severe	*P* value*
Total participants (*N* = 546)	9 (1.6%)	84 (15.4%)	453 (83%)	
Gender (men/women)	8/1	65/19	162/291	** < 0.001**
	Mean (standard deviation)			
Age (years)	56.0 (11.3)	57.5 (10.9)	57.0 (10.9)	0.901
Age at diagnosis of T2DM (years)	38.0 (8.6)	42.0 (11.2)	42.2 (10.7)	0.413
Duration of T2DM (years)	15.8 (8.4)	15.5 (7.3)	14.7 (8.2)	0.487
BMI (kg/m^2^)	22.7 (1.4)	25.4 (2.4)	32.8 (5.6)	** < 0.001**
C-RP (mg/L [< 5 mg/L])	5.0 (7.25)	15.5 (35.50)	17.6 (39.1)	0.107

^*^P value was determined by chi-square and Kruskal‒Wallis tests. *P* < 0.05, significant

### Hepatic Fibrosis Severity Among Emirati Patients with MASLD

Assessment of liver fibrosis in the 546 T2DM Emirati patients by blood-based markers revealed a significantly lower number of patients with moderate/severe fibrosis according to the APRI than according to the FIB-4 index (Tables 4 and 5). While the APRI identified 96% of the patients with mild fibrosis, the FIB-4 index identified only 72%. Although 83% of the cohort exhibited severe steatosis, both the FIB-4 index and APRI identified severe fibrosis in only 2.5% of patients. Demographic characteristics such as sex, age, age at diagnosis of T2DM, and duration of T2DM were not significantly correlated with the severity of fibrosis according to either index. However, liver fibrosis, such as steatosis, seems to increase significantly (*P* < 0.001) with increasing BMI. Pearson’s chi-square was used to compare the distribution of steatosis by FIB-4 and APRI. The chi-square statistic obtained was highly significant (135.7589 and df = 2) (*P* value is < 0.00001). The main difference was in the distribution of mild and moderate cases between FIB-4 and APRI. The sensitivity of FIB-4 was 88% while that of APRI was 12%. On the other hand, the specificity of FIB-4 was 72% while that of APRI was 96%. However, both methods gave the same estimate for the severe form.

**Table 4:** Demographics of 546 T2D Emirati Patients Categorized According to Liver Fibrosis Severity based on the FIB-4 Index

Liver fibrosis severity	Mild	Moderate	Severe	*P* value*
Total participants (*N* = 546)	393 (71.9%)	139 (25.5%)	14 (2.6%)	
Gender (men/women)	157/236	71/68	7/7	0.060
	Mean (± standard deviation)			
Age (years)	53.4 (9.6)	54.1 (10.0)	56.0 (7.5)	0.472
Age at diagnosis of T2DM (years)	39.0 (11.4)	42.5 (11.1)	41.5 (9.4)	0.378
Duration of T2DM (years)	14.4 (7.5)	11.6 (6.8)	14.6 (8.1)	0.305
BMI (kg/m^2^)	27.6 (4)	30.9 (4)	32.5 (5.4)	** < 0.001**
C-RP (mg/L [< 5 mg/L])	17.7 (40.6)	13,8(26.4)	30.9 (64.3)	0.075

^*^P value was determined by chi-square and Kruskal‒Wallis tests. *P* < 0.05, significant

**Table 5:** Demographics of 546 T2D Patients Categorized According to Fibrosis Severity based on APRI

Liver fibrosis severity	Mild	Moderate	Severe	*P* value*
Total participants (*N* = 546)	525 (96.2%)	8 (1.5%)	13 (2.4%)	
Gender (men/women)	222/303	6/2	7/6	0.130
	Mean (± standard deviation)			
Age (years)	57.1 (10.9)	52.6 (11.6)	56.1 (10.4)	0.598
Age at diagnosis of T2DM (years)	42.1 (10.8)	40.5 (8.2)	43.6 (9.5)	0.659
Duration of T2D (years)	15.0 (8.2)	12.1 (8.8)	12.5 (7.9)	0.343
BMI (kg/m^2^)	31.4 (5.9)	35.4 (6.7)	31.5 (7.2)	0.197

^*^P value was determined by chi-square and Kruskal‒Wallis tests. *P* < 0.05, significant

### Correlations Between Serum-Based Noninvasive Tests and Imaging-Based Transient Elastography (TE)

A total of 108 T2DM Emirati patients were randomly selected for TE by FibroScan^®^. The sub-cohort demographics are shown in Table S3. The mean age at diagnosis in the sub-cohort was ~ 41 years, the mean BMI was 30.71 kg/m^2^, the mean fasting blood glucose level was 147.1 (± 45) mg/dL, the mean AST:ALT ratio was 1.29, the mean HbA1c level was 7.3 (± 1.4%), and the mean C-reactive protein was 19.9 mg/L (± 37.3 SD) (< 5 mg/L). No significant sex differences were observed (Table S3). The demographic distribution for steatosis and fibrosis severity determined by FibroScan^®^ for the sub-cohort of 108 T2DM Emirati patients is shown in Table 6. Although the prevalence of moderate/severe steatosis was 69%, only 22% of patients displayed moderate/severe fibrosis. Only age of diagnosis of T2DM and BMI differed significantly between the groups (*P* > 0.05). Other parameters showed no difference. However, patients with mild steatosis were overweight, while patients with moderate/severe steatosis were significantly more likely to be obese (*P* value < 0.001). Nearly 51% of the sub-cohorts were obese, 29% had elevated triglycerides, 9.3% had elevated AST, and 11.2% had elevated ALT. Cardiometabolic risk factors such as high WC were reported in 59% of men (WC > 40 inches, *N* = 30/57) and 86% of women (Table S4).

**Table 6:** Demographics of 108 T2DM Emirati Patients with MASLD Categorized According to Severity Determined by Imaging-Based Transient Elastography (TE)

Steatosis (FibroScan^®^ CAP score)				
	Mild	Moderate	Severe	*P* value*
Total participants (*N* = 108)	34 (31.4%)	19 (17.5%)	55 (50.9%)	
Gender, *N* (men/women)	17/17	9/10	25/30	0.927
	Mean (± standard deviation)			
Age (years)	53.4 (9.6)	54.1 (10.0)	56.0 (7.5)	0.472
Age at diagnosis of T2D (years)	39.0 (11.4)	42.5 (11.1)	41.5 (9.4)	0.378
Duration of T2DM (years)	14.4 (7.5)	11.6 (6.8)	14.6 (8.1)	0.305
BMI (kg/m^2^)	27.6 (4)	30.9 (4)	32.5 (5.4)	** < 0.001**
Fibrosis (FibroScan^®^ LSM)				
	Mild	Moderate	Severe	*P* value*
Total participants (*N* = 108)	86 (79.6%)	12 (11.1%)	10 (9.3%)	
Gender, *N* (men/women)	41/45	5/7	5/5	0.911
	Mean (± standard deviation)			
Age (years)	54.2 (9.1)	59.1 (7.5)	55.9 (5.4)	0.086
Age at diagnosis of T2D (years)	39.7 (10.4)	47.5 (9.7)	43.0 (8.1)	**0.045**
Duration of T2DM (years)	14.4 (7.5)	11.6 (7.2)	12.9 (9.5)	0.400
BMI (kg/m^2^)	30.1 (4.8)	32.3 (5.9)	33.8 (8.1)	0.291

*N*, number of patients; *LSM*, liver stiffness measurements; *CAP score*, controlled attenuation parameter; *T2D*, type 2 diabetes

^*^P value was determined by chi-square and Kruskal‒Wallis tests. *P* < 0.05, significant

**Figure 1:**
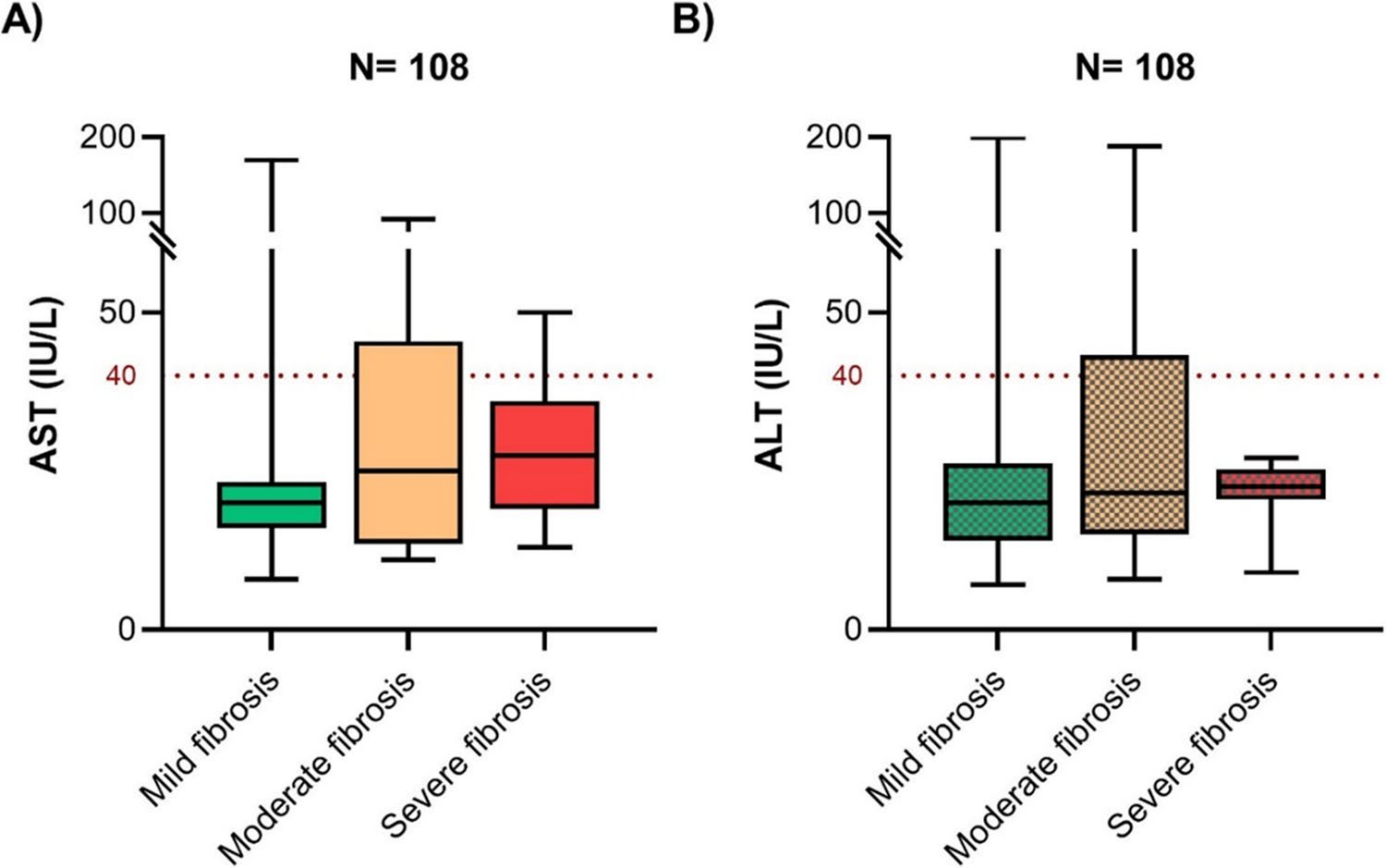
The AST and ALT profiles of the study cohorts. Box plots depicting the variations in **A** AST and **B** ALT across fibrosis severities determined by LSM in the sub-cohort are also depicted as box plots. The Kruskal‒Wallis test showed no significant difference between the severity groups. The dotted horizontal line (red) is the cutoff for elevated AST and ALT levels. For each box plot, the solid lines represent the median, boxes represent the lower and upper quartiles, and whiskers represent the minimum and maximum values.

The liver stiffness measurements of the 108 patients in the T2DM sub-cohort showed that 12 had moderate fibrosis, and only 10 had severe fibrosis. Patients with severe fibrosis were diagnosed with diabetes at an older age than those with mild/moderate fibrosis. Although the ALT level was greater than the AST level in the total cohort, no significant difference was observed in the sub-cohort (Supplementary Fig. 2). Of the 9.3% of patients with elevated AST, 5% had severe fibrosis (according to the LSM), and of the 11.2% of patients with elevated ALT, 8% had severe fibrosis (Fig. 1). No significant differences in the AST/ALT ratio were found between the fibrosis categories.

To assess the relationship between serum biomarkers (FIB-4 and APRI) and imaging-based LSM, the Spearman correlation test was performed (Table 7). The FIB-4 index and APRI showed the greatest correlation between methods (*ρ* = 0.814, *P* value < 0.001). Weakly significant positive correlations were also found between the FIB-4 index and LSM (*ρ* = 0.231, *P* = 0.016), between the APRI and LSM (*ρ* = 0.206, *P* = 0.032), and between the LSM and CAP (*ρ* = 0.262, *P* = 0.006). No significant correlation was detected between the HSI and CAP scores.

**Table 7:** Correlations Between Serum-Based NITs of MASLD and Transient Elastography for the Sub-cohort of 108 Patients

Sub-cohort (*n* = 108)		HSI	APRI	FIB-4 index	LSM	CAP score
HSI	*ρ*	1.000	.145	.284^**^	.187	.187
	Sig. (2-tailed)		.135	.003	.053	.053
	*N*	108	108	108	108	108
APRI	*ρ*	.145	1.000	.814^**^	.206^*^	.031
	Sig. (2-tailed)	.135		< .001	.032	.750
	*N*	108	108	108	108	108
FIB-4 index	*ρ*	.284^**^	.814^**^	1.000	.231^*^	-.016
	Sig. (2-tailed)	.003	< .001		.016	.870
	*N*	108	108	108	108	108
LSM	*ρ*	.187	.206^*^	.231^*^	1.000	.262^**^
	Sig. (2-tailed)	.053	.032	.016		.006
	*N*	108	108	108	108	108
CAP score	*ρ*	.187	.031	-.016	.262^**^	1.000
	Sig. (2-tailed)	.053	.750	.870	.006	
	*N*	108	108	108	108	108

*HSI*, hepatic steatosis index; *APRI*, aspartate aminotransferase-to-platelet ratio index; *FIB-4 index*, fibrosis-4 index; *LSM*, liver stiffness measurements; *CAP score*, controlled attenuation parameter; *N*, number of patients; *ρ*, Spearman’s rank correlation coefficient

^*^Correlation is significant at the 0.05 level (2-tailed). **Correlation is significant at the 0.01 level (2-tailed)

The diagnostic accuracy of the blood-based markers against the TE for both steatosis and fibrosis showed that more positive cases were detected by blood-based tests than without the test, with a very low rate of false positives (Table S5).

## DISCUSSION

In this study, a cohort of 546 Emirati patients with long-standing T2D were screened for liver steatosis using HSI^34^ and for liver fibrosis using the FIB-4 index^41^ and APRI indices.^34^ Approximately 98% of patients showed moderate-to-severe steatosis, which increased proportionately with increasing BMI. Using FIB-4, 20% of patients had moderate-to-severe liver fibrosis, which proportionately increased with increasing BMI. Both the APRI and FIB-4 indices consistently identified patients with severe fibrosis at the ~ 2.5% level. Sex differences were detected for steatosis but not for fibrosis, with more women diagnosed with fatty liver than men. A further sub-cohort of 108/546 patients underwent transient elastography. Using CAP scores, 18% had moderate steatosis, and 51% had severe steatosis. Using LSM, 11% of patients had moderate fibrosis, while 9% had severe fibrosis. Although 51% of the 108 sub-cohort displayed severe steatosis, only 9% had severe liver fibrosis.

The diagnostic technique used in any study significantly impacts the incidence of steatosis in patients with T2DM. This study reported a prevalence of 70% by transient elastography, similar to the findings of Lomonaco et al.^45^ which is higher than that reported by Younossi et al.^24^ based on ultrasound (~ 55%). Moderate-to-advanced fibrosis (F2 or higher), an established risk factor for cirrhosis and overall mortality, affects at least one out of six patients with T2DM. These results support the use of the American Diabetes Association guidelines to screen for clinically significant fibrosis in patients with T2DM with steatosis or elevated ALT.

A significantly high number of patients in our study were identified to have severe fibrosis by FibroScan^®^ (~ 9%). Lomonaco et al.^45^ reported similar results in a study involving 561 T2DM patients in the USA. In the present study, imaging-based measurements of steatosis severity (CAP) were significantly correlated with measurements of fibrosis severity (LSM), as reported by Shah et al.^46^ These findings indicate that the two contribute to the same underlying pathophysiology. The LSM measured by elastography was significantly correlated with the FIB-4 index and APRI. In general, patients with MASLD in this study had higher BMIs, hyperlipidemia, and kidney dysfunction. Patients with moderate/severe fibrosis were older, had a higher BMI, and were diagnosed with T2DM at an older age than those with mild or no fibrosis. The risk of developing MASH increases with increasing BMI.^47^ The positive correlation of BMI with disease severity shows that the Emirati population, which is adversely affected by obesity, is at high risk of developing chronic liver disease. However, it seems that the control of glucose homeostasis in patients with long-term T2DM, such as fasting blood glucose and HbA1c levels, is no longer related to the severity of MASLD. Elevated AST or ALT (≥ 40 units/L) was present in a minority of patients with steatosis (8% and 13%, respectively) or with liver fibrosis (18% and 28%, respectively). This suggests that the AST/ALT ratio alone is insufficient for initial screening. However, performance may be enhanced by imaging (e.g., transient elastography) and plasma diagnostic panels (e.g., FIB-4 and APRI).

NITs for liver fibrosis staging are a major benefit to patients with MASLD. Given its high prevalence, which affects millions of people worldwide, the invasiveness of liver biopsy and sampling errors make it impractical, especially for the periodic assessment required for monitoring disease progression.^48^ NITs allow rapid assessment of large numbers of patients and reliably exclude advanced fibrosis in a high proportion of patients with MASLD (52–62%), allowing liver biopsy to be used in a more direct manner.^49–52^ In many clinics, blood-based markers are the most practical tests widely used. However, most experts consider transient elastography to be the most accurate NIT for identifying Metavir fibrosis of stage > F3, but in clinical practice, it is typically used in conjunction with other indirect or direct measures of hepatic fibrosis.^53^ These factors offer quick and economic benefits to patients with MASLD and the healthcare system, thereby allowing periodic monitoring of disease progression, especially in populations with a high prevalence of risk factors such as obesity and T2DM. Insulin resistance, a key pathophysiological feature of T2DM, plays a central role in the development and progression of MASLD by promoting hepatic lipid accumulation.^54^ Among blood-based tests, the AUROC-measured accuracy of the FIB-4 index is significantly better for diagnosing advanced fibrosis.^47,55,56^ However, this can be affected by the prevalence of obesity among MASLD patients.^55^ Steatosis affects the sensitivity of the FIB-4 index without affecting its specificity in patients with MASLD syndrome.^55^ Similar results have been obtained for LSM in the presence of severe steatosis among patients with advanced fibrosis.^57^

The use of noninvasive fibrosis scoring systems was validated by Önnerhag et al.^58^ in a study with a long-term follow-up time (~ 19 years on average). They also confirmed a significant correlation between the scores and incident DM and cardiovascular disease. The International Clinical Practice Guidelines^43^ recommend screening for MASLD to be part of routine work-up in patients with type 2 diabetes, obesity, and metabolic syndrome. The EASL guidelines^16^ for MASLD management endorse the APRI, FIB-4, and LSM as the most validated tools for the diagnosis of severe fibrosis without a consensus on thresholds or strategies for their application in clinical practice when trying to avoid liver biopsy. Calculating the FIB-4 index and APRI at regular intervals, not only at baseline, will likely increase the predictive capacity. However, this finding indicates the possibility for early assessment of MASLD patients at risk of future complications.^58^

As of 2024, an average of 32.1% of the adult population of the UAE (men, 28.6%; women, 39.4%) is obese, with 91.3% being overweight (men, 71.8%; women, 69.7%) and at risk of obesity.^59^ In other Middle East and North Africa countries, the age-standardized prevalence of obesity in adults increased from 1990 to 2022 for both sexes.^59^ With similar risk factors, a wider screening of associated metabolic diseases such as T2DM, obesity, and MASLD among these countries of similar ethnic origin is warranted.

## Conclusions

This is the first large-scale study of MASLD in the UAE aiming to establish the magnitude of disease burden in random patients with long-term T2DM with the aim of improving noninvasive diagnosis and future management strategies. The concomitant use of imaging (TE) and a blood biomarker panel approach reduces the misdiagnosis of severe fibrosis. The alarming rate of steatosis (≥ F2 > 70%) among Emirati diabetes patients indicates a high risk of severe liver fibrosis in the population. The current study identified one in four participants (20%) with moderate-to-severe fibrosis by transient elastography, which was similar to the results of the blood-based FIB-4 index (≥ F2, 28%) but greater than the APRI (≥ F2, 4).

## Limitations

Several confounding factors may affect the results, such as the accuracy of laboratory parameters, false elevations in LSMs due to acute hepatitis, extrahepatic cholestasis, congestive heart failure, hepatic amyloidosis, and recent food intake. The current study was a one-time study conducted in one clinical center, and the results need to be validated in prospective studies with larger numbers of Emirati T2D patients from all over the UAE with follow-ups.

## Supplementary Information

Below is the link to the electronic supplementary material.

Supplementary file1 (DOCX 138 KB)

## Data Availability

All data analyzed during this study are included in this published article and its supplementary information.

## Declarations:

### Disclaimer:

The views expressed in the submitted article are our own and not an official position of the institution or funder.

## Abbreviations

NAFLD: Nonalcoholic fatty liver disease
MASLD: Metabolic dysfunction–associated steatotic liver disease
T2DM: Type 2 diabetes mellitus
FIB-4: Fibrosis-4
APRI: Aspartate aminotransferase-to-platelet ratio index
HSI: Hepatic steatosis index
LSM: Liver stiffness measurement
CAP: Controlled attenuation parameter
NASH: Nonalcoholic steatohepatitis
NIT: Noninvasive tests
VCTE: Vibration-controlled transient elastography
ADA: American Diabetic Association
GADA: Glutamic acid decarboxylase antibodies
MODY: Maturity-onset diabetes of the young
HbA1c: Hemoglobin A1c
BMI: Body mass index
AST: Aspartate aminotransferase
ALT: Alanine aminotransferase
kPa: Kilopascals
dB/m: Decibels per meter
CI: Confidence interval
SD: Standard deviation
*ρ*: Rho
*χ*^2^: Chi-square
DDC: Dubai Diabetes Center
DSREC: Dubai Scientific Research Ethics Committee
IRB: Institutional review board
PPV: Positive predictive value
NPV: Negative predictive value
ROC: Receiver operating characteristic
