# Supplementary material for: Noninvasive Assessment of the Severity of Liver Fibrosis in MASLD Patients with Long-Standing Type 2 Diabetes
Source: J Gen Intern Med. 2025 Jan 22;40(10):2309–18. doi: 10.1007/s11606-025-09348-2 (PMC12344024; doi:10.1007/s11606-025-09348-2)
Supplement: Supplementary file 1 — Supplementary file1 (DOCX 138 KB) [file 11606_2025_9348_MOESM1_ESM.docx]

**APPENDICES:**

**Supplementary table 1: Clinical features of the patient cohort (N=546).**

|  | **All** | **Men** | **Women** | **P value (adj)** |
| --- | --- | --- | --- | --- |
| Number of patients (N) | 546 | 235 | 311 | -- |
| Age at visit (years) | 57.63 (10.86) | 57.34 (10.44) | 57.85 (11.17) | >.999 |
| Age at diagnosis of T2DM (years) | 42.15 (10.72) | 41.82 (10.65) | 42.4 (10.79) | >.999 |
| Duration of T2DM (years) | 15.45 (8.19) | 15.45 (8.65) | 15.45 (7.84) | >.999 |
| BMI (kg/m^2^) | 31.44 (6.06) | 29.97 (5.34) | 32.55 (6.34) | >.999 |
| Waist:Hip ratio | 0.9685 (0.08) | 0.9982 (0.06) | 0.9443 (0.08) | >.999 |
| Systolic BP (mmHg) | 131.4 (14.51) | 131.4 (15.81) | 131.4 (13.48) | >.999 |
| Diastolic BP (mmHg) | 72.87 (11.28) | 75.76 (11.18) | 70.69 (10.86) | >.999 |
| Pulse (b/m) | 81.14 (11.93) | 79.32 (11.34) | 82.52 (12.19) | >.999 |
| ALT (IU/L) | 24.65 (19.89) | 28.83 (23.14) | 21.49 (16.38) | >.999 |
| AST (IU/L) | 21.93 (14.48) | 22.75 (14.06) | 21.31 (14.79) | >.999 |
| AST:ALT ratio | 1.091 (0.79) | 0.9683 (0.61) | 1.183 (0.90) | >.999 |
| HDL (mg/dL) | 47.86 (12.54) | 43.36 (9.667) | 51.26 (13.38) | >.999 |
| LDL (mg/dL) | 83.6 (32.25) | 82.26 (32.3) | 84.61 (32.22) | >.999 |
| Triglycerides (mg/dL) | 135.3 (75.9) | 135 (68.72) | 135.6 (81.02) | >.999 |
| Total Cholesterol (mg/dL) | 158.3 (37.87) | 152.2 (37.66) | 162.9 (37.44) | >.999 |
| FBG (mg/dL) | 144.6 (52.68) | 143.7 (47.64) | 145.2 (56.24) | >.999 |
| HbA1c (%) | 7.616 (1.641) | 7.552 (1.614) | 7.664 (1.661) | >.999 |
| Blood Urea (mg/dL) | 31.68 (18.44) | 34.34 (21.52) | 29.66 (15.45) | >.999 |
| Serum Creatinine (mg/dL) | 0.7821 (0.41) | 0.9204 (0.42) | 0.6775 (0.36) | >.999 |
| eGFR (mL/min) | 94.46 (21.23) | 92.75 (21.25) | 95.75 (21.17) | >.999 |
| Urine albumin (mg/L) | 82.98 (283.1) | 110.7 (383.9) | 59.15 (146.6) | **<.001** |
| Urine creatinine (mg/dL) | 105 (76.31) | 123.8 (83.34) | 90.28 (66.83) | **0.025** |
| Albumin/Creatinine Ratio (mg/g) | 126.3 (512.5) | 118.1 (507.3) | 133 (517.6) | 0.985 |
| Albumin Blood (g/dL) | 4.23 (0.34) | 4.31 (0.31) | 4.169 (0.35) | >.999 |

T2DM- Type 2 diabetes mellitus; BMI- body mass index; BP- blood pressure; ALT- alanine aminotransferase; AST- aspartate aminotransferase; HDL- high-density lipoprotein; LDL- low-density lipoprotein; FBG- fasting blood glucose; HbA1c- hemoglobin A1c; eGFR- estimated glomerular filtration rate.

All values are shown as the mean (±standard deviation). P values were adjusted for sex effects by applying Šídák's multiple comparisons test with 2-way ANOVA.

**Supplementary table 2: Elevated clinical features of the patient cohort (N=546)**

|  | **Reference** | **Number of patients, N (%)** |
| --- | --- | --- |
| ALT | ≥40 IU/L | 66 (12.1) |
| AST | ≥40 IU/L | 29 (5.4) |
| Obese BMI | ≥40 IU/L | 301 (55.2) |
| LDL | ≥100 mg/dL | 144 (26.4) |
| Triglyceride | ≥150 mg/dL | 169 (31) |
| Total cholesterol | ≥200 mg/dL | 79 (14.5) |
| FBG | ≥125 mg/dL | 319 (58.5) |
| HbA1c | ≥7% | 336 (61.6) |

ALT- alanine aminotransferase; AST- aspartate aminotransferase; BMI- body mass index; HDL- high-density lipoprotein; LDL- low-density lipoprotein; FBG- fasting blood glucose; HbA1c- hemoglobin A1c.

**Supplementary table 3: Clinical features of the patient subcohort (N=108).**

|  | **All** | **Men** | **Women** | **P value (adj)** |
| --- | --- | --- | --- | --- |
| Number of patients (N) | 108 | 51 | 57 |  |
| Age at visit (years) | 55.32 (8.667) | 55.06 (7.778) | 55.56 (9.455) | >.999 |
| Age at diagnosis of T2DM (years) | 40.91 (10.33) | 40.33 (9.591) | 41.42 (11.01) | >.999 |
| Duration of T2DM (years) | 14.42 (7.672) | 14.73 (7.595) | 14.14 (7.798) | >.999 |
| BMI (kg/m2) | 30.71 (5.386) | 29.45 (4.987) | 31.82 (5.524) | >.999 |
| Waist:Hip ratio | 0.9726 (0.07) | 1.002 (0.04) | 0.9459 (0.07) | >.999 |
| Systolic BP (mmHg) | 128.3 (12.7) | 128 (14.17) | 128.5 (11.34) | >.999 |
| Diastolic BP (mmHg) | 73.67 (9.8) | 74.65 (9.773) | 72.79 (9.827) | >.999 |
| Pulse (b/m) | 80.25 (11.78) | 78.39 (11.48) | 81.96 (11.89) | >.999 |
| ALT (IU/L) | 26.31 (26.54) | 30.04 (26.21) | 22.98 (26.62) | >.999 |
| AST (IU/L) | 26.59 (25.84) | 26.25 (22.94) | 26.89 (28.38) | >.999 |
| AST:ALT ratio | 1.296 (1.481) | 1.054 (1.014) | 1.512 (1.781) | >.999 |
| HDL (mg/dL) | 48.75 (12.66) | 43.08 (9.366) | 53.82 (13.12) | >.999 |
| LDL (mg/dL) | 81.8 (31.54) | 78.61 (31.76) | 84.65 (31.34) | >.999 |
| Triglycerides (mg/dL) | 133.3 (90.25) | 139.4 (80.58) | 127.7 (98.48) | >.999 |
| Total Cholesterol (mg/dL) | 156 (37.1) | 147.9 (36.13) | 163.3 (36.75) | >.999 |
| FBG (mg/dL) | 147.1 (45.46) | 148.4 (44.01) | 145.9 (47.09) | >.999 |
| HbA1c (%) | 7.335 (1.437) | 7.445 (1.67) | 7.237 (1.197) | >.999 |
| Blood Urea (mg/dL) | 28.28 (8.465) | 30.14 (8.697) | 26.61 (7.964) | >.999 |
| Serum Creatinine (mg/dL) | 0.762 (0.51) | 0.8412 (0.20) | 0.6912 (0.68) | >.999 |
| eGFR (mL/min) | 99.27 (18.17) | 98.25 (15.63) | 100.2 (20.28) | >.999 |
| Urine albumin (mg/L) | 28.58 (48.64) | 31.78 (41.64) | 25.46 (54.93) | >.999 |
| Urine creatinine (mg/dL) | 103.4 (69.85) | 120 (72.78) | 87.76 (63.73) | 0.966 |
| Albumin/Creatinine Ratio (mg/g) | 90.13 (548.9) | 25.48 (30.19) | 152.1 (766.4) | **<.001** |
| Albumin Blood (g/dL) | 4.281 (0.31) | 4.375 (0.25) | 4.196 (0.33) | >.999 |
| CRP (mg/L [<5mg/L]) | 19.9 (37.3) | 21.2 (40.4) | 18.8 (35.1) | 0.107 |

T2DM- Type 2 diabetes mellitus; BMI- body mass index; BP- blood pressure; ALT- alanine aminotransferase; AST- aspartate aminotransferase; HDL- high-density lipoprotein; LDL- low-density lipoprotein; FBG- fasting blood glucose; HbA1c- hemoglobin A1c; eGFR- estimated glomerular filtration rate; CRP- C-Reactive Protein.

All values are shown as the mean (±standard deviation). P values were adjusted for sex effects by applying Šídák's multiple comparisons test with 2-way ANOVA.

**Supplementary table 4: Elevated clinical features of the patient cohort (N=108)**

|  | **Reference** | **Number of patients, N (%)** |
| --- | --- | --- |
| ALT | ≥40 IU/L | 12 (11.2) |
| AST | ≥40 IU/L | 10 (9.3) |
| Obese BMI | ≥30 kg/m2 | 55 (51) |
| LDL | ≥100 mg/dL | 25 (23.2) |
| Triglyceride | ≥150 mg/dL | 31 (28.8) |
| Total cholesterol | ≥200 mg/dL | 14 (13) |
| FBG | ≥125 mg/dL | 68 (63) |
| HbA1c | ≥7% | 66 (61.2) |
| WC- Men | ≥35 inch | 47 (92.2) |
| WC-Women | ≥40 inch | 30 (52.7) |

ALT- alanine aminotransferase; AST- aspartate aminotransferase; BMI- body mass index; HDL- high-density lipoprotein; LDL- low-density lipoprotein; FBG- fasting blood glucose; HbA1c- hemoglobin A1c.

**Supplementary table 5: Diagnostic Serum-based indices against Transient Elastography (FibroScan®)**

| **N** | | | **Total** | **Sensitivity** | **Specificity** | **PPV** | **NPV** |
| --- | --- | --- | --- | --- | --- | --- | --- |
| **FIB-4 index** | | | | | | | |
| **LSM** | F0 & F1 | F2, F3 & F4 | Total | (14/22)  63.6% | (59/86)  68.6% | (14/41)  34.1% | (59/67)  88.1% |
| F0 & F1 | 59 | 27 | 86 |  |  |  |  |
| F2, F3 & F4 | 8 | 14 | 22 |  |  |  |  |
| Total | 67 | 41 | 108 |  |  |  |  |
| **APRI** | | | | | | | |
| **LSM** | F0 & F1 | F2, F3 & F4 | Total | 3/22)  13.6% | (81/86)  94.2% | (3/8)  37.5% | (81/100)  81% |
| F0 & F1 | 81 | 5 | 86 |  |  |  |  |
| F2, F3 & F4 | 19 | 3 | 22 |  |  |  |  |
| Total | 100 | 8 | 108 |  |  |  |  |
| **HSI** | | | | | | | |
| **CAP** | Grade 0 & 1 | Grade 2 & 3 | Total | (74/75)  98.7% | (2/33)  6.1% | (74/105)  70.5% | (2/3)  66.7% |
| Grade 0 & 1 | 2 | 31 | 33 |  |  |  |  |
| Grade 2 & 3 | 1 | 74 | 75 |  |  |  |  |
| Total | 3 | 105 | 108 |  |  |  |  |

PPV: positive predictive value; NPV: negative predictive value; HSI- hepatic steatosis index; APRI- aspartate aminotransferase-to-platelet ratio index; FIB-4 index- fibrosis-4 index; LSM- liver stiffness measurements; CAP score- controlled attenuation parameter; N- number of patients.


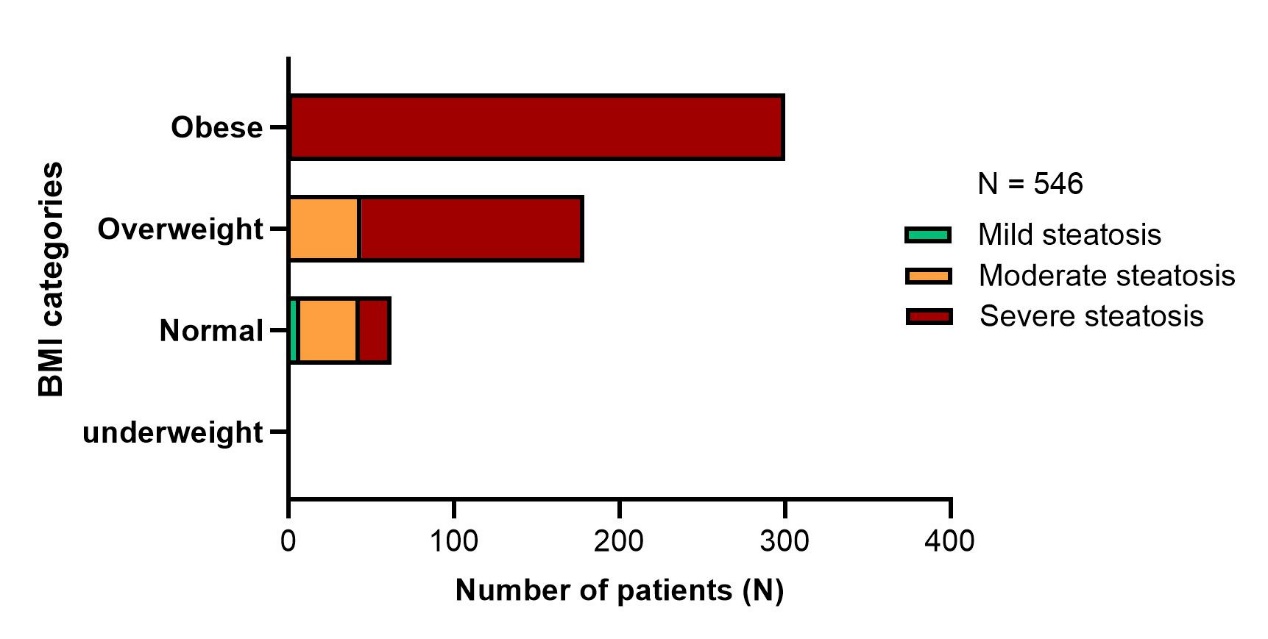


**Supplementary Fig. 1:** **BMI distribution of the study cohort and their subclassification based on steatosis severity determined by the hepatic steatosis index (HSI).** The number of patients (N) in each category is plotted along the X axis. BMI was classified as underweight: <18.5 kg/m^2^; normal: ≥18.5-25 kg/m^2^; overweight: ≥25-30 kg/m^2^; and obese ≥30 kg/m^2^.


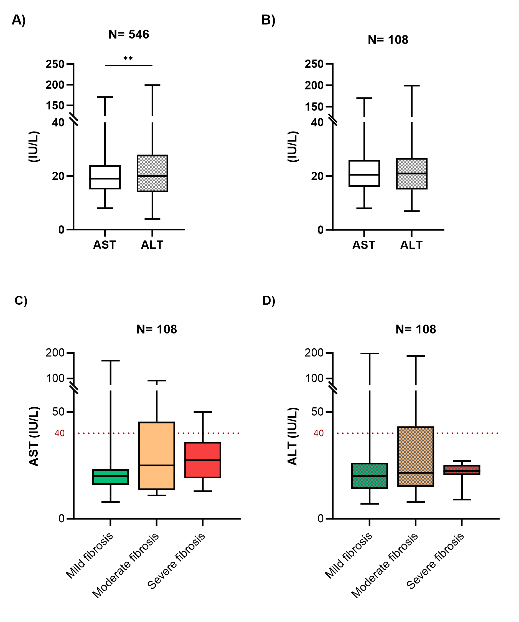


**Supplementary Fig. 2:** **AST and ALT profiles of the study cohorts.** Box plots depicting the overall AST and ALT levels in the **A)** study cohort (n= 546) and **B)** subcohort (n= 108). Statistical differences between two parameters were determined by paired t tests with two-tailed significance. For each box plot, the solid lines represent the median, boxes represent the lower and upper quartiles, and whiskers represent the minimum and maximum values
